# Utility of a Digital PCR-Based Gene Expression Panel for Detection of Leukemic Cells in Pediatric Acute Lymphoblastic Leukemia

**DOI:** 10.3390/ijms27020674

**Published:** 2026-01-09

**Authors:** Jesús García-Gómez, Dalia Ramírez-Ramírez, Rosana Pelayo, Octavio Martínez-Villegas, Lauro Fabián Amador-Medina, Juan Ramón González-García, Augusto Sarralde-Delgado, Luis Felipe Jave-Suárez, Adriana Aguilar-Lemarroy

**Affiliations:** 1 Programa de Doctorado en Ciencias Biomédicas, Centro Universitario de Ciencias de la Salud, Universidad de Guadalajara, Guadalajara 44340, Mexico; jesus.garcia9891@alumnos.udg.mx; 2 Laboratorio de Oncoinmunología y Citómica del Cáncer Infantil, Centro de Investigación Biomédica de Oriente, Instituto Mexicano del Seguro Social, Puebla 74360, Mexico; josefina.ramirez@imss.gob.mx (D.R.-R.); rosana.pelayo@imss.gob.mx (R.P.); 3 Unidad de Educación e Investigación, Instituto Mexicano del Seguro Social, Mexico City 06725, Mexico; 4 Unidad Médica de Alta Especialidad, Hospital de Gineco-Pediatría No. 48, Centro Médico Nacional del Bajío, León 37328, Mexico; octavio.martinez@ugto.mx; 5 Unidad Médica de Alta Especialidad, Hospital de Especialidades No. 1, Centro Médico Nacional del Bajío, León 37320, Mexico; lauro.amador@imss.gob.mx; 6 División de Genética, Centro de Investigación Biomédica de Occidente (CIBO), Instituto Mexicano del Seguro Social (IMSS), Guadalajara 44340, Mexico; jrgg_gene@hotmail.com; 7 Coordinación Auxiliar Médica de Investigación en Salud, OOAD Jalisco, Instituto Mexicano del Seguro Social (IMSS), Guadalajara 44340, Mexico; augusto.sarralde@imss.gob.mx; 8 División de Inmunología, Centro de Investigación Biomédica de Occidente (CIBO), Instituto Mexicano del Seguro Social (IMSS), Guadalajara 44340, Mexico

**Keywords:** leukemia, ALL, *JUP*, *NT5C3B*, *GATA3*, *PTK7*, *MYC*, *CNP*, *SNAI1*, *ICOSLG*

## Abstract

Acute lymphoblastic leukemia (ALL) is a genetically heterogeneous disease where current clinical practice guidelines remain focused on traditional cytogenetic markers. Despite recent advances demonstrating excellent diagnostic accuracy for gene expression signatures, a discontinuity exists between biomarker validation and clinical implementation. This study aimed to develop and validate a multiparametric gene expression signature using digital PCR (dPCR) to accurately diagnose pediatric ALL, with potential utility for monitoring measurable residual disease (MRD). We analyzed 130 bone marrow aspirates from pediatric patients from four clinical groups: non-leukemia, MRD-negative, MRD-positive and leukemia characterized by immunophenotype. Gene expression of an 8-gene panel (*JUP*, *MYC*, *NT5C3B*, *GATA3*, *PTK7*, *CNP*, *ICOSLG*, and *SNAI1*) was quantified by dPCR. The diagnostic performance of individual markers was assessed, and a Random Forest machine learning model was trained to classify active disease. The model was validated using a 5-fold stratified cross-validation approach. Individual markers, particularly *JUP*, *MYC*, and *NT5C3B*, showed good diagnostic accuracy for distinguishing leukemia from non-leukemia. However, integrating all eight markers into a multivariate Random Forest model significantly enhanced performance. The model achieved a mean cross-validated area under the curve (AUC) of 0.908 (±0.041) on receiver operator characteristic (ROC) analysis and 0.961 (±0.019) on Precision–Recall (PR) analysis, demonstrating high reliability and a favorable balance between sensitivity and precision. The integrated model achieved high sensitivity (88.9%) for detecting active disease, particularly at initial diagnosis. Although specificity was moderate (65.0%), the high positive predictive value (PPV 85.1%) and accuracy (81.5%) confirm the clinical utility of a positive result. While the panel showed promising performance for distinguishing MRD-positive from MRD-negative samples, the limited MRD-positive cohort size (n = 11) indicates that validation in larger MRD-focused studies is required before clinical implementation for treatment monitoring. This dPCR-based platform provides accessible, quantitative detection without requiring knowledge of clonal shifts or specific genomic landscape, offering potential advantages for resource-limited settings such as those represented in our Mexican pediatric cohort.

## 1. Introduction

Pediatric Acute Lymphoblastic Leukemia (ALL) is the most common childhood cancer worldwide [1]. Although detection and management protocols in well-resourced and developed regions of the world have achieved 5-year survival rates of over 90%, children from less privileged backgrounds—such as those in low- and middle-income countries like Mexico—often experience significantly lower survival rates, frequently below 60% [2,3,4].

Given the heterogeneity of the disease, it requires morphologic, immunophenotypic, cytogenetic, and molecular studies for accurate classification and risk stratification. Diagnosing ALL involves challenges related to early disease detection, accurate phenotyping, and risk stratification. A fundamental obstacle in leukemia treatment is delayed diagnosis, which occurs when the symptoms are mistaken for benign conditions. Furthermore, in resource-limited countries, diagnosing ALL presents additional technical difficulties due to limited access to specialized tests such as multiparametric flow cytometry, genomic sequencing, and molecular testing. Conventional bone marrow staining for morphological analysis may underestimate leukemic burden in hypocellular cases or failures in the bone marrow aspirate sampling.

The current gold standard for diagnosis and monitoring relies on immunophenotyping, a flow cytometry-based approach that identifies and characterizes leukemic cells using selectively expressed antigens [5,6]. However, flow cytometry can yield false-negative results when ALL cells display atypical phenotypes and low marker expression or when the sensitivity of the test does not exceed the required limits. This technique also requires extensive antibody panels, expertise in identifying aberrant phenotypes, and adequate cellularity.

In this context, molecular tests have increasingly been proposed and integrated into ALL clinical workflows to provide an additional layer of precision. For instance, the use of reverse transcription quantitative polymerase chain reaction (RT-qPCR), digital PCR (dPCR), and next-generation sequencing (NGS) are now used to detect gene fusion transcripts [7,8,9], immunoglobulin/T-cell receptor (IG/TCR) rearrangements [10], somatic mutations [11], and aberrantly expressed genes [12,13]. These methods have proven effective in complementing—and in some cases, surpassing—the sensitivity of immunophenotyping assessments.

Another major challenge in ALL management is Measurable Residual Disease (MRD), which significantly impacts prognosis. Detecting residual leukemic cells after treatment is difficult with conventional methods (e.g., morphological microscopy), necessitating highly sensitive techniques such as multiparameter flow cytometry, PCR, or NGS. MRD monitoring has prognostic value, as MRD positivity correlates with a higher early relapse risk. Patients with persistent MRD exhibit lower disease-free survival (DFS) and overall survival (OS). Consequently, MRD-guided therapy—adjusting treatment intensity based on MRD results—has been proposed to improve outcomes.

In a previous study of our research group [14], we identified a set of genes located within common chromosomal gains in ALL patients and leukemia-derived cell lines, validating their expression through microarray and RNA sequencing analyses. Among these, *JUP* (Junction Plakoglobin), *NT5C3B* (5-Prime-nucleotidase, cytosolic IIIB), and *CNP* (Cyclic Nucleotide Phosphodiesterase), emerged as clinically relevant candidates. Notably, hierarchical clustering revealed strikingly high expression levels of *JUP* in B-ALL patients, as well as in hematopoietic stem cells (HSCs), but not in other normal hematopoietic lineages. Importantly, *JUP* overexpression was strongly associated with poor overall survival in B-ALL, underscoring its potential prognostic value.

This work further assesses the utility and potential of *JUP*, *NT5C3B*, and *CNP* as novel biomarkers for leukemia and EMR detection. Bone marrow aspirates from pediatric patients were included in this study and analyzed using reverse transcription followed by dPCR. The expression level of each gene was classified according to immunophenotyping-based diagnosis. Additionally, previously reported leukemic cell biomarkers like *MYC* (MYC Protooncogene, bHLH Transcription Factor), *PTK7* (Protein-tyrosine kinase 7), *GATA3* (GATA-binding protein 3), *ICOSLG* (Inducible T-cell costimulator ligand), and *SNAI1* (SNAIL family transcriptional repressor 1) were included for comparison.

## 2. Results

### 2.1. Clinical and Demographic Characterization of the Study Groups

A total of 178 bone marrow aspirate samples were collected, processed, and analyzed for gene expression. To ensure that gene expression analysis was not biased by RNA quality, samples were filtered applying a dPCR threshold of a minimum of 10,000 positive partitions for at least one housekeeping gene. Of the 178 samples initially collected, 48 were excluded for failing to meet this quality criterion. These exclusions were distributed as follows: non-leukemia (n = 7), MRD-negative (n = 11), MRD-positive (n = 4), and leukemia (n = 26). This step ensured that subsequent gene expression analysis was performed on samples with adequate RNA quality and sufficient template for reliable quantification. The final cohort consisted of 130 samples: 11 individuals without leukemia (non-leukemia controls with non-malignant hematological conditions requiring bone marrow evaluation to exclude malignancy, detailed in Section 4), 29 samples that were MRD-negative during follow-up, 11 samples that were MRD-positive during follow-up (≥0.01% blast cells), and 79 samples that were positive for ALL at diagnosis.

**Figure 1:**
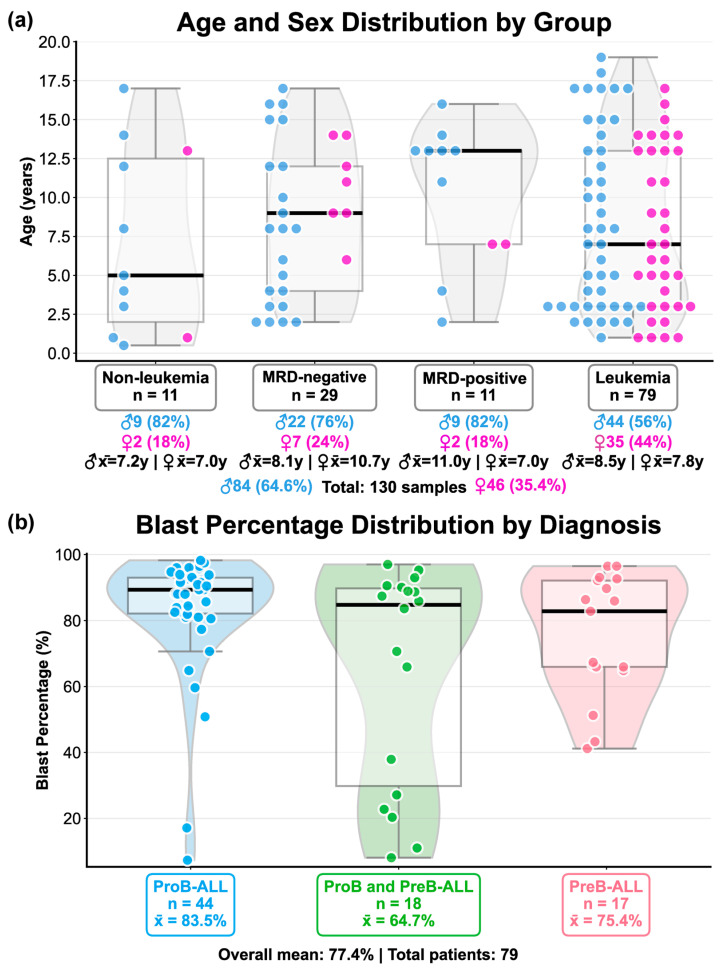
Clinical and demographic characteristics of the study cohorts: (**a**) Age distribution by study group and sex. Groups represent the continuum of the disease as follows: Non-leukemia (controls free from leukemia), minimal residual disease (MRD)-negative (patients in remission with undetectable MRD), MRD-positive (patients undergoing remission induction or maintenance treatment with detectable MRD), and Leukemia (patients with acute lymphoblastic leukemia, ALL, at diagnosis). Violin plots show the density of age distribution within each group; internal boxplots show the interquartile range (median lines). Each colored dot represents an individual observation (blue: male; pink: female). For each group, the following demographic data are summarized below: sample size (n), sex distribution (%), and mean ages ($\bar{X}$), stratified by sex. (**b**) Distribution of blast percentages across immunophenotypic subtypes in the leukemia group. Subtypes are ProB-ALL, ProB-PreB-ALL and PreB-ALL group. Each violin contour shows the density of blast percentages within each leukemic subtype; internal boxplots show the interquartile range with median indicated by a black line. Each colored dot represents an individual measurement. Below each group, the sample size (n) and mean blast percentage ($\bar{X}$) are displayed.

Analysis of demographic data indicated a male predominance across all study groups (64.6%; 84/130) of the total samples (Figure 1a). The Non-leukemia, MRD-negative, and MRD-positive groups were predominantly male (82%, 76%, and 82%, respectively). In contrast, the leukemia group showed a more equitable male-to-female ratio (56% vs. 44%). The mean age was relatively consistent across all groups, with the male MRD-positive cohort having the highest median age (11 years) and the non-leukemia group the lowest (7.0 years). In the largest group (leukemia), the mean age was 8.5 years for males and 7.8 years for females.

As visualized in Figure 1b, analysis of blast percentage in the ALL-diagnosis group (n = 79) revealed an overall mean of 77.4%. The results stratified by subtype were as follows: ProB-ALL was the most prevalent subtype (n = 44) and exhibited the highest mean blast percentage (83.5%). The PreB-ALL subtype (n = 17) showed a mean blast percentage of 75.4%, while the mixed ProB and PreB-ALL group (n = 18) had the lowest mean blast percentage (64.7%).

Additionally, the distribution of immunophenotypic subtypes across all the clinical groups is shown in Supplementary Figure S1. For MRD samples, the subtype classification corresponds to the original diagnostic immunophenotype when available. ProB-ALL was the most frequent subtype across all groups (leukemia: 55.7%, MRD-positive: 63.6%, MRD-negative: 41.4%). Notably, no mixed ProB/PreB-ALL phenotype cases were observed in the MRD-positive group. All samples were included in the downstream analyses regardless of their subtype.

### 2.2. Comparative Analysis of Gene Expression Divergence Across Study Groups

**Figure 2:**
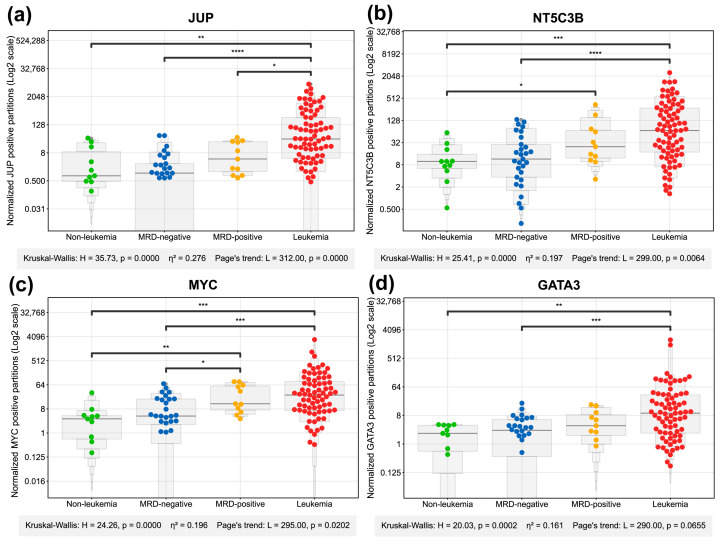
Digital PCR (dPCR) analysis of target genes expression across patient groups. Swarm plots display the normalized positive partition counts for *JUP* (**a**), *NT5C3B* (**b**), *MYC* (**c**), and *GATA3* (**d**) measured in bone marrow samples from different diagnostic categories: Non-leukemia controls (green), MRD-negative (blue), MRD-positive (yellow), and leukemia patients (red). Each dot represents an individual sample measurement. Gene expression was normalized using *RPS18* and *RPLP0* as reference genes. The y-axis shows uses a log2 scale to optimally visualize the widely dispersed expression values. Note that zero values cannot be displayed on logarithmic scale. The gray enhanced box plot contours display letter-value summaries, with box widths proportional to the square root of the number of observations at each level. Statistical information below each panel includes: Kruskal–Wallis H value, *p*-value for overall group differences, eta-squared (η^2^) effect size, and Page’s L trend statistic with corresponding *p*-value to evaluate monotonic trends across the disease progression groups. Post hoc Mann–Whitney U test significance is indicated by horizontal bars with asterisk notation: * (*p* < 0.05), ** (*p* < 0.01), *** (*p* < 0.001), **** (*p* < 0.0001). The absence of a bar denotes a non-significant result. Effect sizes for Kruskal–Wallis tests were interpreted according to Cohen’s guidelines: negligible (η^2^ < 0.01), small (0.01 ≤ η^2^ < 0.06), medium (0.06 ≤ η^2^ < 0.14), and large (η^2^ ≥ 0.14).

**Figure 3:**
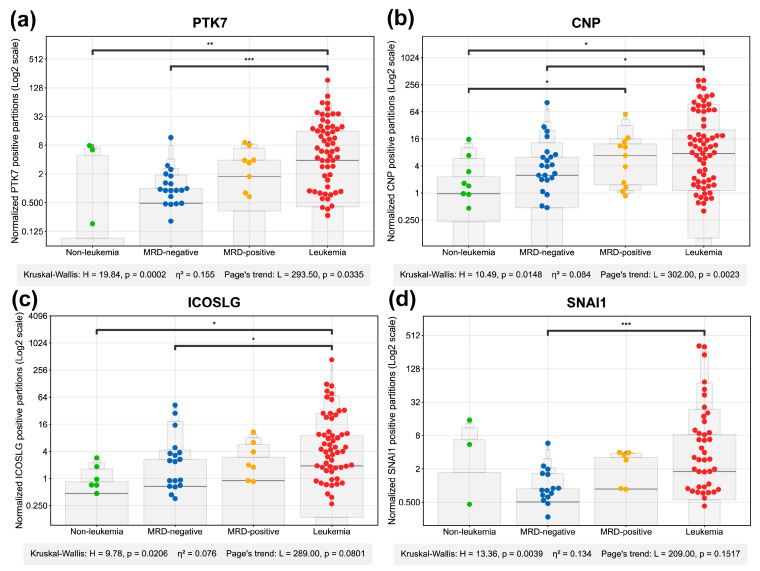
Digital PCR analysis of target genes expression across patient groups. Swarm plots display the normalized positive partition counts for *PTK7* (**a**), *CNP* (**b**), *ICOSLG* (**c**) and *SNAI1* (**d**) measured in bone marrow samples from different diagnostic categories: Non-leukemia controls (green), MRD-negative (blue), MRD-positive (yellow), and leukemia patients (red). Post hoc Mann–Whitney U test significance is indicated by horizontal bars with asterisk notation: * (*p* < 0.05), ** (*p* < 0.01), *** (*p* < 0.001). The absence of a bar denotes a non-significant result. A description of additional data similar, provided in the same format as the caption for Figure 2.

To assess the clinical value of the proposed gene panel, gene expression quantification at transcriptional level using dPCR assays and dedicated probes was performed for all study groups. As depicted in Figure 2 and Figure 3, high expression of *JUP*, *NT5C3B*, *MYC*, *GATA3*, *PTK7*, *CNP*, and *ICOSLG* clearly differentiated the leukemia from the non-leukemia group. Statistically significant differences between the leukemia and MRD-negative groups were achieved by *JUP*, *NT5C3B*, *MYC*, *GATA3*, *PTK7*, *CNP*, *SNAI1*, and *ICOSLG*. High expressions of *NT5C3B*, *MYC,* and *CNP* were also able to differentiate between the non-leukemia and MRD-positive groups, while only *MYC* could distinguish between MRD-negative and MRD-positive groups. Importantly, *JUP*, *NT5C3B*, *MYC*, and *GATA3*, *PTK7*, *CNP*, and *ICOSLG* differentiated both non-pathological conditions (non-leukemia and MRD-negative) from leukemia samples.

In contrast, *BIRC5*, a classic gene expression marker that has been reported in many malignancies including ALL [15,16], did not reveal statistically significant differences in any test. Therefore, in the context of this study, it would not offer any clinical value.

**Figure 4:**
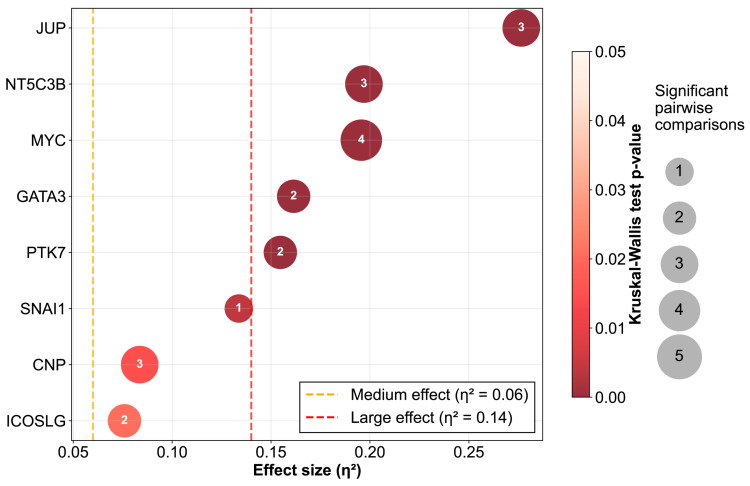
Comprehensive statistical summary of differential gene expression across study groups. Each bubble represents an individual gene from the panel. The bubble size corresponds to the number of statistically significant pairwise comparisons (Mann–Whitney U tests, *p* < 0.05) between clinical groups. Color intensity represents the Kruskal–Wallis (KW) *p*-values, where darker red indicates strongest statistical significance (lower *p*-values); non-significant results displayed in gray. The x-axis shows the eta-squared (η^2^) effect size from KW test, representing the proportion of variance in gene expression explained by group membership. Effect sizes are interpreted according to Cohen’s guidelines as: negligible (η^2^ < 0.01), small (0.01 ≤ η^2^ < 0.06), medium (0.06 ≤ η^2^ < 0.14), and large (η^2^ ≥ 0.14).

A statistical summary of these comparisons is presented in Figure 4, where each marker is represented as a bubble, whose color, size and position encode the effect size, Kruskal Wallis (KW) significance, and the number of significant pairwise comparisons. Notably, *JUP*, *NT5C3B*, *MYC*, *GATA3*, and *PTK7* emerged as the features with higher differences between groups. These genes exhibited large effect sizes (η^2^ > 0.14) and high statistical significance, indicating a substantial and robust differential expression between the patient groups. *SNAI1*, *CNP,* and *ICOSLG* also showed significant changes but with more moderate effect sizes.

### 2.3. Diagnostic Performance of Individual Gene Expression

We next evaluated the diagnostic potential of candidate markers for their ability to accurately classify positive and negative classes. To this end, we performed receiver operating characteristic (ROC) analyses across four clinically relevant comparisons: (1) leukemia vs. non-leukemia (emulating a primary diagnosis scenario); (2) leukemia + MRD-positive vs. MRD-negative + non-leukemia (to assess overall disease activity); (3) MRD-positive vs. MRD-negative (to evaluate the discrimination of MRD among treated patients); and (4) leukemia vs. MRD-negative + non-leukemia (challenging the gene panel to distinguish the leukemia disease group from a combined group of patients who are either negative for MRD or do not have leukemia). The area under the curve (AUC) constitutes an important metric for representing the probability that a randomly selected positive case will have a higher gene expression level than a randomly selected negative case. The discriminatory capacity of a model based on its AUC is typically interpreted as follows: poor (<0.70), acceptable/fair (0.70–0.79), good (0.80–0.89), or excellent (≥0.90). Due to the uncertainty in AUC estimation introduced by comparisons with small sample sizes, bootstrap confidence intervals (CIs) were calculated to provide a robust quantification without relying on distributional assumptions.

**Figure 5:**
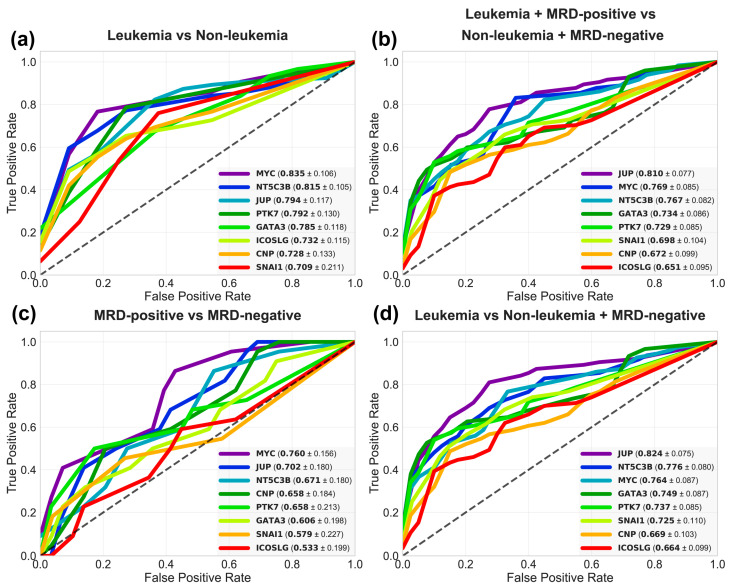
Receiver operating characteristics (ROC) analysis of gene expression biomarkers for leukemia diagnosis and monitoring. ROC curves depict the diagnostic performance of nine candidate gene expression markers across relevant clinical comparisons; (**a**) Leukemia vs. non-leukemia (n = 90) for initial diagnosis; (**b**) Leukemia + MRD-positive vs. non-leukemia + MRD-negative (n = 130) for detection of overall disease activity; (**c**) MRD-positive vs. MRD-negative (n = 40) for monitoring measurable residual disease; (**d**) Leukemia vs. MRD-negative + non-leukemia (n = 119) for distinguishing leukemia patients from remission and non-cancerous states. Individual markers are labeled with their area under the curve (AUC) and corresponding bootstrap 95% confidence intervals (±) from 1000 iterations. The dotted line represents the performance of a random classifier (AUC = 0.5).

In the first comparison (Figure 5a), which addresses the primary diagnosis question, *MYC* demonstrated the highest performance (AUC = 0.835), followed closely by *NT5C3B* (AUC = 0.815), *JUP* (AUC = 0.794), *PTK7* (AUC = 0.792), and *GATA3* (AUC = 0.785). These results indicate good diagnostic capacity and strong reliability, as evidenced by the low uncertainty in the performance estimations (CI ≤ 0.13). In the second comparison (Figure 5b), which represents the largest cohort, the top markers showed consistent but moderately reduced overall performance. *JUP* led with an AUC of 0.810, followed by *MYC* (AUC = 0.769) and *NT5C3B* (AUC = 0.767). Notably, uncertainty in this analysis was lower (CI ≤ 0.10). The third comparison (Figure 5c) represents the most challenging discrimination: detecting MRD among only treated patients. This analysis showed higher uncertainty (CI > 0.20), and the lowest discriminatory power among individual markers, which were led by *MYC* (AUC = 0.760), and *JUP* (AUC = 0.702). The modest AUCs combined with wide confidence intervals and small sample size (MRD-positive n = 11, MRD-negative n = 29) indicate that these findings are exploratory. Finally, the comparison of leukemia cases at diagnosis against non-leukemia + MRD-negative individuals (Figure 5d) yielded similar results to the first comparison. *JUP* was the top performer (AUC = 0.824), followed by *NT5C3B* (AUC = 0.776), and *MYC* (AUC = 0.764).

Additional to the individual marker performance assessment across multiple clinical scenarios, we conducted a comprehensive evaluation of optimal diagnostic cutoffs and clinical metrics for each marker using the most relevant comparison: active disease vs. absence of disease (leukemia and MRD-positive patients versus non-leukemia and MRD-negative patients, n = 130). We identified the cutoff value that yielded the highest Youden index, which reflects an optimized balance between sensitivity and specificity. *PTK7* emerged as the best-balanced marker, with the highest Youden index of 0.520. At an optimal cutoff of 3.41 normalized positive partitions, it achieved 65.7% sensitivity and 86.4% specificity, resulting in 70.8% overall accuracy and an excellent positive predictive value (PPV) (0.936). *JUP* ranked second, with a Youden index of 0.491. It demonstrated 69.1% sensitivity and 80.0% specificity at a cutoff of 11.25 normalized units, yielding 71.9% accuracy and 90.6% positive predicted value. *MYC* achieved a Youden index of 0.463, with a notably high sensitivity of 84.5% but a reduced specificity of 61.8% at its optimal cutoff value of 5.12. This provided 78.0% accuracy and the highest negative predictive value (NPV) of 61.8%. *GATA3* followed with a Youden index of 0.433. It showed a modest sensitivity of 50.0% but excellent specificity of 93.3%, corresponding to the highest PPV of 95.5% and a lower overall accuracy of 61.4% at a threshold of 9.00. The remaining biomarkers showed moderate performance with Youden indices ranging from 0.366 to 0.422: *NT5C3B* (sensitivity 62.2%, specificity 80.0%), *SNAI1* (sensitivity 58.7%, specificity 83.3%), *ICOSLG* (sensitivity 55.6%, specificity 83.3%), and *CNP* (sensitivity 56.6%, specificity 80.0%).

### 2.4. Diagnostic Performance of a Multivariate Gene Panel Integration

To determine if a gene panel provides better diagnostic performance than individual biomarkers, we developed a Random Forest machine learning model. This model was trained using gene expression patterns of all markers demonstrating diagnostic potential: *JUP*, *MYC,* *NT5C3B*, *SNAI1*, *GATA3*, *PTK7*, *CNP* and *ICOSLG*. *BIRC5* was excluded due to a lack of significant expression differences between groups and negligible discrimination power. The model was trained to classify samples into active disease (ALL-positive at diagnosis or MRD-positive during follow-up) from those without the disease (non-leukemia or MRD-negative during follow-up). This approach allowed us to include all samples and address the key clinical challenge of detecting disease traces from initial diagnosis through treatment monitoring.

**Figure 6:**
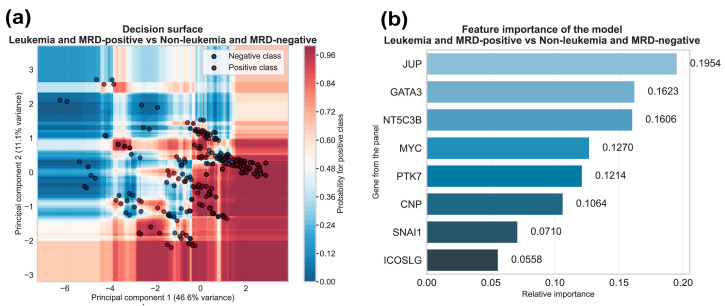
Multivariate Random Forest analysis for the detection of ALL across the disease continuum: (**a**) Decision surface plot in principal component space showing individual patient observations as dots (red: ALL-positive and MRD-positive; blue: ALL-negative and MRD-negative). These are overlaid on a color-graded area representing the predicted likelihood of active disease at any principal component analysis (PCA) coordinate, demonstrating the model’s decision boundaries to effectively separate active disease from its absence using the 8-gene panel signature of each patient. Principal components 1 and 2 explain 45.0% and 12.6% of the variance, respectively; (**b**) Relative feature importance analysis, estimating of each gene’s contribution to the model’s discriminatory power, calculated by measuring the mean decrease in Gini impurity across all 100 decision trees in the Random Forest model, with *JUP*, *GATA3* and *NT5C3B* emerging as the top contributors.

First, to demonstrate the algorithm capacity to learn meaningful patterns from the gene expression data across our groups, we trained the model on the complete dataset. The decision surface plot, projected onto the principal component space via principal component analysis (PCA), visualizes this learning. In this plot, individual observations are shown as points (red represents the positive class, active disease at the time of diagnosis or during follow-up; blue represents the negative class, disease-free/remission). These observations are distributed over an area coded with a colored gradient, indicating the model’s predicted probability of belonging to the positive class at any given PCA coordinate. The visualization reveals a clear separation between the positive and negative classes in distinct clusters, with most patients correctly positioned in regions matching their predicted class probabilities. This pattern confirms that the machine learning model successfully identified discriminatory gene expression signatures within the 8-gene panel (Figure 6a).

**Figure 7:**
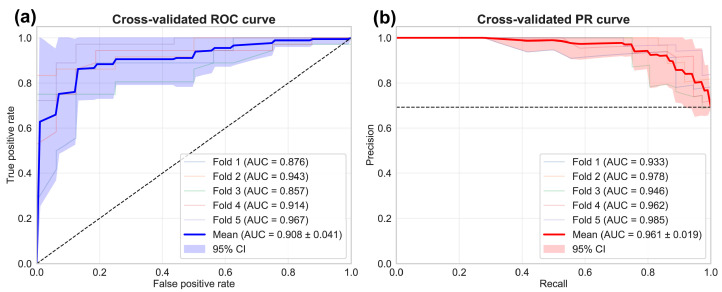
5-fold cross-validation evaluation of the Random Forest (RF) model’s performance: (**a**) ROC curves from 5-fold stratified cross-validation. Each patient was evaluated by a model that was not trained on their data, providing an unbiased assessment of the 8-gene panel’s clinical utility. Individual fold performance is shown as light-colored lines with their corresponding AUC values. The mean performance is depicted by the dark blue line (AUC = 0.908 ± 0.041), and the 95% confidence interval is represented by the shaded blue area. The diagonal dashed line indicates the performance of a random classifier. (**b**) Precision–recall (PR) curves from the same 5-fold cross-validation approach. Individual fold performances are shown as light-colored lines with their AUC values. The mean performance is shown as a red line, with the 95% confidence interval as shaded red area. The horizontal dashed line represents the baseline performance of a random classifier, given the prevalence of positive cases in the dataset.

Complementarily, we performed a relative feature importance (RFI) analysis to quantify how much each individual feature contributes to improving node purity of all 100 decision trees across the Random Forest model. This metric (range: 0.0–1.0) reflects both the frequency a marker is selected for splitting nodes in the decision trees and the effectiveness of those splits in discriminating between patients with leukemia and disease-free patients. This analysis identified *JUP* as the most important contributor to the model’s performance (RFI = 0.1954), followed by *GATA3* (RFI = 0.1623) and *NT5C3B* (RFI = 0.1606) (Figure 7b). The middle-tier contributors included *MYC* (RFI = 0.1270), *PTK7* (RFI = 0.1214), and *CNP* (RFI = 0.1064), while *SNAI1* (RFI = 0.0710) and *ICOSLG* (RFI = 0.0558) showed the lowest contributions. Notably, the top three markers (*JUP*, *GATA3*, and *NT5C3B*) collectively accounted for over half (51.83%) of the panel’s total discriminative power (Figure 6b).

Subsequently, to evaluate the model’s true performance in an unbiased manner, we performed a 5-fold stratified cross-validation. In this process, the data were randomly divided into five splits while preserving the proportion of positive and negative cases in each fold. For each of the five iterations, four splits were used as the training cohort and the remaining one as the test cohort. This rigorous approach ensured that predictions for each patient were generated by a model that had not been trained on that patient’s data, thus providing unbiased performance estimates for our 8-gene panel in a real-world clinical scenario.

For each of the five iterations, we calculated individual ROC curves and their corresponding area under the curve (AUC), ranging from 0.857 to 0.967. The results were averaged to obtain a mean cross-validated AUC of 0.908 ± 0.041 (Figure 7a). The consistently high AUC values across all folds demonstrate the robustness of our 8-gene panel in discriminating active disease from the absence of leukemic blasts, with minimal variation between different patient subsets.

To complement the ROC analysis and provide a more exhaustive evaluation of our model’s performance, we also generated precision–recall (PR) curves using the same cross-validation approach. While ROC analysis reveals the trade-off between sensitivity (true positive rate) and the false positive rate across all classification thresholds, PR curves focus on the relationship between precision (PPV) and recall (true positive rate, sensitivity). This offers a performance perspective centered on the accuracy of the positive prediction. The AUC values for the PR curves ranged from 0.933 to 0.985, with a mean AUC of 0.961 ± 0.019 (Figure 7b). The sustained high precision across most recall values demonstrates that the model achieves high predictive accuracy while successfully capturing most true positive cases. In clinical context, this indicates that the 8-gene panel can reliably identify active disease states without generating excessive false alarms. The narrow confidence interval further indicates high reproducibility across different patient subsets.

Importantly, these results demonstrate superior performance of the integrated panel compared to any individual marker, as shown in Figure 5b. This underscores the critical advantage of multivariate disease characterization over the quantification of individual markers for detecting leukemic signatures across the entire disease spectrum.

**Figure 8:**
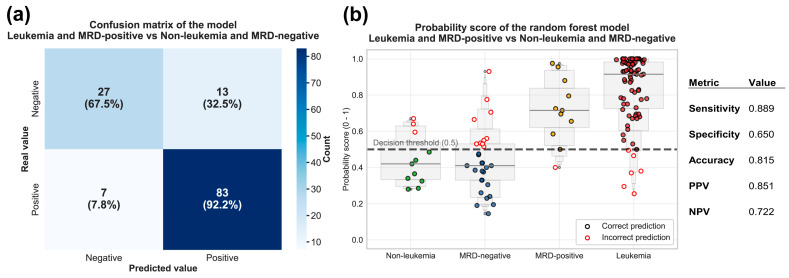
Cross-validated performance metrics and probability score distribution of the 8-gene Random Forest model. (**a**) Confusion matrix based on cross-validated predictions for each of the patients. The matrix compares actual versus predicted classifications. Values show patient counts with row-wise percentages. Key performance metrics are provided, including sensitivity (88.9%), specificity (65.0%), accuracy (81.5%), as well as positive (85.1%) and negative (72.2%) predictive values; (**b**) Swarm plot showing individual patient probability scores generated via 5-fold cross-validation, stratified into four clinical groups: non-leukemia, MRD-negative, MRD-positive, and leukemia. Correct predictions are depicted as solid-colored points, whereas misclassifications are highlighted with red outlines. The horizontal dashed line indicates the 0.5 decision threshold.

To further provide clinically relevant performance metrics, we generated a confusion matrix using cross-validated probability scores from the Random Forest model. Among the 90 patients with active disease, 83 (92.2%) were correctly classified as positive, while (7.8%) were misclassified as disease-free. Conversely, among the 40 individuals in remission or without the disease, 27 (67.5%) were correctly classified as negative, whereas 13 (32.5%) were incorrectly classified as having active disease (Figure 8a). The model demonstrated high sensitivity (88.9%), with performance predominantly driven by newly diagnosed cases. However, the moderate specificity (65%) indicates that while over half of the disease-free patients were correctly identified, a substantial proportion were not. The PPV was 85.1% and the NPV was 72.2%. The moderate specificity suggests that some negative patients, particularly those with prediction scores near the decision threshold, may benefit from additional confirmatory testing to rule out false positives. These cross-validated metrics provide a realistic approximation of how the gene panel would behave in an unknown clinical population.

Finally, to visualize the model’s performance across the disease spectrum in our cohort, we plotted the cross-validated predicted probability scores for individual patients, stratified by their clinical status. As shown in Figure 8b, each point represents an individual patient’s Random Forest probability score (ranged 0–1). A decision threshold of 0.50 is shown with correct predictions depicted as solid circles and incorrect predictions highlighted with a red outline.

The plot reveals distinct clustering patterns aligned with disease activity. Patients with leukemia at diagnosis showed the highest probability scores, with most samples clustering above 0.80 and extending to near-perfect confidence scores (>0.90). In contrast, ALL-negative individuals exhibited lower scores, predominantly clustering below 0.50. The two patient groups monitored for MRD showed overlapping but distinguishable distributions centered around the 0.50 decision threshold. MRD-negative patients generally had scores below 0.5, though with notable variability, while MRD-positive patients typically display higher probabilities above this threshold (Figure 8b). Notably, most misclassifications occurred within the 0.40 to 0.60 probability range, predominantly among MRD-negative patients. This suggests that patients with borderline scores may benefit from additional clinical monitoring. Overall, this visualization demonstrates the model’s high confidence in clear-cut clinical scenarios and its ability to appropriately reflect uncertainty in transitional disease states.

## 3. Discussion

Acute lymphoblastic leukemia is a genetically and biologically heterogeneous disease, and although current clinical practice guidelines remain focused on traditional cytogenetic markers, recent advances in expression profiling have demonstrated excellent diagnostic accuracy for gene expression signatures. This research has provided robust evidence for clinical utility of gene expression markers, with large-scale validation studies revealing a strong diagnostic potential. However, significant discontinuity persists between biomarker validation and implementation as well as regulatory approval. This gap may be partly attributable to the inaccessibility of the techniques employed, since most of these classifiers require time- and resource- consuming techniques like microarray or RNA-Seq assays, followed by complex computational analysis.

For example, Brady et al. in their 2023 study identified 376 driver genes that enable the classification of novel, genomically defined subtypes in childhood ALL patients characterized by cryptic chromosomal rearrangements by transcriptome sequencing [17]. Similarly, Li et al., in their 2009 publication, established a 62-gene classifier with 97.6% accuracy and 97.0% precision, which successfully categorized six major ALL subtypes using microarray data [18]. Other studies have proposed gene expression panels for the prediction of relapse-free survival, like the 38-gene expression classifier derived from TARGET Phase I and II published by Kang et al. [19], which distinguished between low-risk and high-risk pediatric ALL groups using microarray data. In a more recent approach, the ALLCatchR RNA-seq classifier represents the most comprehensive validation effort to date. This machine learning-based tool, trained on transcriptome sequencing data from 1869 patients, allocates B-cell precursor ALL into specific molecular subtypes with 96.7% accuracy across three independent validation cohorts [20]. Collectively, these approaches successfully underscore the clinical utility of gene expression profiling to capture disease heterogeneity through multivariate classification algorithms, often optimized using machine learning models. However, they are not yet widely available for the general pediatric ALL population.

This implementation gap between research validation and clinical adoption creates both challenges and opportunities for gene expression panel development.

Digital PCR has emerged as a method of choice for applying gene expression markers in pediatric ALL, offering several technical advantages. This application has been established as a superior technology for monitoring pediatric ALL by IG/TCR, improving the sensitivity of MRD quantification by a factor of ten (one log_10_) over traditional qPCR methods and resolving 83% of previously inconclusive results [10]. However, a key limitation of this approach is even though IG/TCR rearrangements are present in over 95% of patients [21], they require individualized assay design and continuous monitoring to account for clonal evolution [22].

Furthermore, dPCR’s increased sensitivity, particularly for rare targets, has enabled its application beyond traditional gene fusion monitoring. A recent study published in 2024 successfully designed dPCR assays for 11 rare fusion transcripts in hematological malignancies, achieving detection limits below 0.01% [23]. Fusion transcripts derived from chromosomal rearrangements are highly informative and prognostically valuable in pediatric ALL. This is especially true for classic aberrations, such as *BCR::ABL1*, *ETV6::RUNX1*, *KMT2A::AFF1*, *TCF3::PBX1* and *SIL::TAL1* [24]. The clinical utility of these markers is constrained by their detectability, which is limited to approximately 20 to 30% of pediatric patients; in addition, their expression also varies widely across different ethnicities [3,25]

Our study successfully developed and validated an 8-gene dPCR panel, integrated with a Random Forest model, for detecting across the disease continuum. To our knowledge, this is the first multiparametric gene expression classifier developed for pediatric ALL that uses accessible and quick molecular technology like dPCR to support both diagnosis and disease monitoring. Our approach addresses the unmet need for a universal diagnostic signature applicable to the full spectrum of pediatric ALL cases. It provides an accessible and rapid platform that simultaneously overcomes the dependence on patient-specific genomic rearrangements.

The 8-gene expression panel (*JUP*, *CNP*, *NT5C3B*, *ICOSLG*, *SNAI1*, *PTK7*, *MYC*, and *GATA3*) we validated captures a transcriptional profile encompassing diverse oncogenic pathways, cell survival mechanisms, and immune regulatory processes. This integrated dPCR assay enables absolute quantification of target transcripts without standard curves, a known limitation of qPCR, and delivers a robust test applicable to a wide range of patients, independent of their individual genomic landscape, potentially offering advantages for resource-limited settings like our context. These capabilities are key to addressing the translational gap, moving gene expression markers from a research context into an accessible, dPCR-based clinical diagnostic.

An important consideration is that our non-leukemia control group consists of pediatric bone marrow samples with various non-malignant hematological conditions, including hemophagocytic lymphohistiocytosis, immune thrombocytopenia, and viral-associated cytopenias, rather than samples from truly healthy marrow. While these underlying conditions may influence baseline gene expression patterns through inflammatory or immune-mediated pathways, this reflects the usual clinical challenge: diagnostic gene panels must differentiate leukemia from other conditions requiring bone marrow evaluation. Importantly, our panel’s ability to distinguish leukemic samples even against this background of non-malignant hematological abnormalities demonstrates its clinical robustness and specificity for detecting leukemia-associated transcriptional signatures. Thus, we consider that the use of these “real-world control samples” comes out as an important strength for the clinical applicability of our findings, as they represent the actual diagnostic dilemmas encountered in pediatric hematology practice.

One of our main limitations is that, while our study design encompasses the continuum from diagnosis through treatment monitoring, the data provides stronger evidence for the panel’s utility in initial disease detection. The comparison of MRD-positive against MRD-negative samples yielded modest AUCs with wide confidence intervals (Figure 5c), reflecting both the small sample size and the inherent difficulty of detecting low-level residual disease through gene expression profiling. Furthermore, in our Random Forest classifier, most “active disease” cases were newly diagnosed leukemia (79/90 samples), meaning the model’s performance metrics are dominated by overt disease detection rather than sensitive MRD discrimination. Hence, we must acknowledge that this class imbalance limits the machine learning model’s ability to learn MRD-specific discriminatory signatures.

To validate added value of the Random Forest model, we compared its performance against a logistic regression baseline using the same gene panel and cross-validation strategy (as observed in Supplementary Table S1 and Supplementary Figure S2). The Random Forest model demonstrated superior performance, with an AUC of 0.908 ± 0.041 and a sensitivity of 88.9%, compared to the logistic regression’s AUC of 0.821 ± 0.033 and sensitivity of 61.1%. This improvement likely stems from the Random Forest’s capacity to capture non-linear relationships and gene interaction effects that are inaccessible to linear models.

The current data primarily supports the panel’s role as an adjunct diagnostic tool at initial presentation and as a general “active disease” detector. The MRD-related findings should be considered exploratory and require validation in larger, dedicated MRD cohorts with prospective designs, including serial samples from individual patients and correlation with clinical outcomes, to determine whether this gene expression approach can achieve the performance required for MRD-guided therapeutic decisions. An additional consideration is that MRD classification was determined by multiparameter flow cytometry with 10^−4^ sensitivity, and discordances between flow cytometry and gene expression may occur near this threshold, as the methods measure different biological signals. Future studies should investigate such borderline cases and include comparison with additional MRD detection methods.

An additional limitation was the choice of imputation method for technically missing values. Multivariate imputation by chained equations (MICE) with Bayesian Ridge regression is one of several valid approaches and could influence subtle performance differences. This is particularly relevant for *SNAI1*, which was the only one with a high proportion of missing data (25.38%), and for interpretation of low-expressed genes in MRD contexts, where transcript abundance is inherently near the detection threshold.

In this study, *JUP* emerged as one of the top markers for its individual performance for the detection of active disease, with an AUC of 0.810 (Figure 5b). It also made the strongest contribution to the classifier, with a relative feature importance value of 0.1954 (Figure 6b). The selection of this marker is supported by previous studies from our group, which linked chromosome 17 copy number gains in ALL samples and cell lines to *JUP* overexpression of up to 46.9-fold; this overexpression was correlated with worse survival outcomes [14]. Our comparative genomic hybridization (CGH) analysis identified chromosomes 14, 17, and 22 as containing the most frequent copy gains, with chromosome 17 being the most prevalent. A common gain region at 17q21.2 encompasses *JUP*, *NT5C3B* and *CNP*. Independent databases validation confirmed the recurrence of this alteration. Furthermore, studies in mouse models and patient-derived samples have shown that *JUP* acts as an enabling factor for disease initiation and progression of *BCR::ABL1*-positive B-ALL in an *MYC*-dependent manner by augmenting and enforcing *BIRC5* expression [26]. Elevated levels of *JUP* have also been detected in prostate cancer, early-stage ovarian cancer [27], and in acute myeloid leukemia patients [28].

*GATA3* demonstrated strong clinical potential, achieving the second highest relative feature importance (0.1623, Figure 6b). Its contribution as an individual marker was less remarkable, with AUCs below 0.80. This suggests that *GATA3* captures a unique portion of the variance not explained by other top markers like *JUP*, *NT5C3B*, *PTK7*, and *MYC.* Consequently, in combination with the top marker *JUP*, *GATA3* contributes more significantly to the multiparametric classification model that it could alone. The relevance of *GATA3* in ALL is supported by previous research linking its germline variants to an up to 2-fold increase in disease susceptibility, particularly among Hispanic patients with Ph-like B-cell ALL [29]. Furthermore, this gene is of particular interest due to its connection with *MYC* in a core oncogenic network, driving T-cell ALL pathogenesis through a *NOTCH1-GATA3-MYC* regulatory cascade [30]. *GATA3* acts as a master chromatin regulator that amplifies *MYC* transcription by recruiting SWI/SNF complexes to create accessible enhancer regions. Recent studies in Mexican pediatric B-ALL patients have demonstrated that *GATA3* germline variants (rs3824662 and rs3781093) are associated not only with disease susceptibility and *CRLF2* overexpression but also correlate with overall survival outcomes [31]. These findings further support *GATA3*′s clinical relevance as both a diagnostic and prognostic marker in pediatric ALL populations of Mexican ancestry.

The *MYC* proto-oncogene is a master regulator of cell growth, proliferation, and metabolism. Its dysregulation is a hallmark of cancer, driving aggressive disease, therapeutic resistance, and poor prognosis [32]. MYC protein expression has been directly correlated with poor overall survival in adult ALL patients and proposed as a prognostic marker, with its expression also being significantly associated with p53 expression in immunohistochemistry assays [33]. In our study, *MYC*’s contribution as an individual marker was strong, achieving the highest AUC for the discrimination between leukemia vs. non-leukemia samples (AUC = 0.835, Figure 5a), and second for leukemia + MRD-positive vs. non-leukemia + MRD-negative samples (AUC = 0.769, Figure 5b). However, its contribution to the machine learning-based classifier was unexpectedly diminished, ranking fourth in relative feature importance (Figure 6b). This suggests that *MYC* accounts for a proportion of the variance that overlaps with *JUP*, this collinearity means *MYC* contributes less independently to the model’s decision boundaries than other key markers, such as *GATA3* and *NT5C3B*.

*NT5C3B* represents a key target discovery with a mechanistic function in regulating nucleotide metabolism and mRNA processing. Despite its significant clinical potential, ranking third in relative feature importance (0.1606, Figure 6b) and demonstrating strong individual diagnostic performance between groups (AUC = 0.815, Figure 5a; and AUC = 0.767, Figure 5b), *NT5C3B* remains largely unexplored in pediatric ALL and in cancer overall. Building on our previous identification of *NT5C3B* within the chromosome 17 gain region, this marker emerged as one of the most promising candidates from our CGH and expression validation studies, with overexpression in B-cell and T-cell ALL patients compared with healthy subjects determined using microarray expression data from the Microarray Innovations in LEukemia (MILE) project [14].

The connection of *NT5C3B* to nucleotidase activity provides a compelling mechanistic rationale for its role in ALL. Cytosolic nucleosidases catalyze dephosphorylation of nucleoside 5′-monophosphates, thereby regulating intracellular nucleotide levels. This enzyme family is critical for ALL outcomes and treatment response; for instance, *NT5C2* mutations drive chemotherapy resistance in early relapse ALL by enhancing nucleotidase activity [34]. Additionally, alternative splicing of *NT5C2* have proven to produce gain-of-function isoforms that contribute to thiopurine resistance [35]. As a member of the same 5′-nucleotidase family, *NT5C3B*, preferentially dephosphorylates pyrimidine nucleotides (CMP, UMP), and less efficiently, purine nucleotides (AMP); it has a unique affinity for hydrolyzing 7-methylguanosine monophosphate (m7GMP) to 7-methylguanosine and inorganic phosphate. This substrate specificity suggests *NT5C3B* prevents undesired accumulation and salvage of m7GMP, potentially impacting cell survival by regulating mRNA turnover and preventing the accumulation of potentially toxic metabolites [36]. The combination of genomic and clinical evidence positions *NT5C3B* as a key target in pediatric ALL, a finding underscored by its outstanding, and previously unrecognized, diagnostic performance. In addition, its established biochemical function in nucleotide metabolism makes a compelling case for prioritizing *NT5C3B* in functional validation studies related to cancer and cell survival. Furthermore, recently developed *NT5C3B* inhibitors [36] present potential therapeutic opportunities.

The protein tyrosine kinase-7 (*PTK7*) is a pseudokinase receptor that regulates cell signaling, polarity, and migration. While crucial for embryogenesis, it is overexpressed in various cancers, such as colon carcinoma, acute and chronic myeloid leukemia, and ovarian cancer, making it a promising therapeutic target [37,38]. *PTK7* demonstrated optimal sensitivity-specificity balance as an individual marker for clinical decision making, achieving the highest Youden index (0.520), as depicted in Table 1. *PTK7* is expressed on hematopoietic cell, its expression is tightly regulated during the maturation of normal thymic T-cells. However, this regulation is disrupted in T-ALL. As demonstrated by Jian et al., *PTK7* is significantly overexpressed in T-cell blasts compared to mature T-cells in human bone marrow, establishing its utility for detecting T-cell MRD [39]. In addition, Li et al., reported that *PTK7* functions as a downstream target of BCL11B, where its knockdown inhibits proliferation and induces apoptosis via TRAIL/p27 upregulation [40]. Altogether, this marker shows established utility for T-ALL immunophenotyping and MRD detection, with emerging CAR-target applications in other malignancies [41]. Although most of these findings in ALL are limited to the T-cell subtype, our findings underscore the clinical value of *PTK7* for the detection of pediatric ALL, both as an individual marker and as a component of the multiparametric mRNA expression panel.

*ICOSLG* is among the markers included in the panel with the weakest discriminative power. Nevertheless, we identified statistically significant differences, evidenced by a significant KW test (*p* = 0.0206) with a moderate effect size (η^2^ = 0.076), as well as a statistically significant pairwise comparison between the leukemia and non-leukemia groups. Although its contribution to the panel was less pronounced than that of stronger markers, this gene has a sound clinical and mechanistic rational. It functions as an immune checkpoint molecule expressed by lymphoid and tumor cells [42]. The interaction between ICOSLG and its receptor, ICOS, promotes the development of T regulatory cells in healthy bone marrow as well as in different tumor microenvironments [43]. Previous studies have reported that elevated *ICOSLG* expression was associated with inferior event-free survival in a cohort of 43 children with t (4;11)-positive ALL and has even been proposed as a therapeutic target [44]. Mechanistically, *ICOSLG* is thought to contribute to immune evasion by promoting the development of T regulatory cells, which suppresses anti-leukemic immune responses. This can allow leukemic cells to persist and re-emerge as relapse despite ongoing chemotherapy [44]. Although the importance of *ICOSLG* should not be overlooked, it is evident that many of the markers identified in our previous genomic studies through chromosome gain analysis surpass previously reported gene expression markers.

*SNAI1* is a key modulator of epithelial-to-mesenchymal transition (EMT) and has been linked to leukemia pathophysiology by impairing differentiation and enhancing self-renewal and proliferation of blast cells through its interaction with the histone demethylase KDM1A/LSD1 [45]. In our study, however, its contribution to the multiparametric model was weak, and its performance as an individual diagnostic marker was among the lowest. Similarly to *ICOSLG*, *SNAI1* expression still revealed statistically significant differences, with a significant KW test (*p* = 0.0039) with a moderate effect size (η^2^ = 0.134), as well as a significant pairwise comparison between MRD-negative and positive patients at diagnosis.

*BIRC5*, commonly known as *SURVIVIN*, is a gene recognized for its strong pan-cancer prognostic value and pro-oncogenic activity in a wide variety of malignancies, particularly in lung cancer [46]. However, its validation as a biomarker for diagnosis and monitoring in pediatric ALL remains limited. In this study, we determined that its expression may not be a reliable tool for clinical disease evaluation, since we do not identify consistent and specific overexpression patterns in samples with leukemic blasts compared to disease-free samples. In one of the first studies that evaluated *BIRC5* expression in bone marrow samples using qPCR, the researchers reported positive expression in more than half of the ALL samples (8 out of 13), while the majority of chronic lymphocytic leukemia (CLL) cases (20 out of 21) exhibited strong expression [47]. On the other hand, a study on a cohort of Egyptian acute leukemia patients—using mononuclear cells from venous blood—reported significantly higher *SURVIVIN* expression in leukemic patients than in controls. It is important to note, however, that this assay was performed with conventional PCR followed by agarose gel electrophoresis, a method that only provides a qualitative detection (presence or absence) of the amplified product [48].

From a holistic functional perspective, this panel aims to capture interconnected oncogenic pathways that collectively address a variety of cancer hallmarks. For instance, the *NOTCH1-GATA3-MYC-BIRC5* has been characterized in T-cell ALL and acute myeloid leukemia, where it drives initiation and progression by coordinating apoptosis resistance [15], chromatin remodeling [30], and transcriptional control [49]. Specifically, *GATA3* can initiate this cascade by functioning as the master chromatin regulator that amplifies *MYC* transcription by recruiting SWI/SNF complexes to create accessible enhancer regions [30].

A parallel network, consisting of *Wnt-JUP-MYC-BIRC5* axis, operates in BCR::ABL1-positive B-cell ALL, where *JUP* acts as a key driver required for leukemia maintenance [26]. While other genes like *PTK7* might be contribute independently by promoting cell survival and chemotherapy resistance through independent pseudo-kinase signaling [50].

The mechanistic diversity captured by the panel supports its clinical utility as a comprehensive gene profiling, monitoring a wide range of cancer-related biological processes including proliferation control, survival signaling, immune evasion and therapeutic resistance. This integrated approach addresses a fundamental limitation of current molecular diagnostic strategies in pediatric ALL, like the dependence on patient-specific genomic rearrangements that are subject to clonal evolution, as well as the detection of fusion genes that are absent in a significant proportion of patients, particularly across ethnically diverse populations. By targeting ubiquitously perturbed transcriptional pathways rather than specific genomic events, our dPCR-based panel provides a novel solution that maintains diagnostic accuracy despite underlying genomic heterogeneity.

Most significantly, the superior performance of a unified multiparametric classifier over individual markers demonstrates the clinical value of feature-diverse strategies for discriminating continuum states in a highly heterogeneous disease like pediatric ALL. The synergistic contribution of markers like *JUP* (Wnt signaling), *GATA3* (chromatin remodeling), *NT5C3B* (nucleotide metabolism), and *PTK7* (cell survival) suggests that leukemic transformation involves coordinated dysregulation across these pathways. This mechanistic complementarity, combined with the accessibility and quantitative precision of dPCR technology, positions our panel as a practical tool with strong potential for implementing precision medicine in clinical practice. Its utility for MRD monitoring, while promising, requires validation in larger treatment-monitoring cohorts.

Finally, as a proof-of-concept study, our work focused on demonstrating clinical discriminatory capacity and did not include formal analytical validation of the dPCR assay. Consequently, metrics such as intra- and inter-assay variability, reproducibility across runs and operators, linearity, and systematic determination of the limit of detection and quantitation for each target gene were not assessed. Furthermore, while the normalization strategy ensured adequate RNA quality, it was established empirically. Comprehensive analytical validation is therefore a prerequisite for the future clinical implementation of this gene expression panel.

## 4. Materials and Methods

### 4.1. Sample Collection, Pre-Processing and Ethical Considerations

Bone marrow aspirate samples were obtained from two independent centers: the Childhood Cancer Oncoimmunology and Cytomics Laboratory from the Eastern Biomedical Research Center of the Mexican Social Security Institute (IMSS) in Puebla, Mexico, and from the High Specialty Medical Unit #48 for Women’s and Children’s Care of the IMSS in Guanajuato, Mexico. Multiparameter flow cytometry using the EuroFlow panel was utilized for the classification and detection of leukemia and MRD. B-ALL was classified into three categories based on CD34 expression and cell lineage: ProB-ALL (CD34^+^ CD19^+^), ProB-PreB-ALL (CD34^−/+^ CD19^+^), and PreB-ALL (CD34^-^ CD19^+^) [51]. EMR samples were stratified into two groups: MRD-negative (undetectable MRD) for those with a blast percentage < 0.01%, corresponding to the analytical sensitivity threshold of 10^−4^, and MRD-positive (detectable MRD) for those with ≥ 0.01% blasts [52].For each patient, written informed consent was obtained from parents or legal tutors, with additional assent provided by the minors themselves. The research protocol was approved by the IMSS Ethics and Research Committees, and CONBIOETICA-09-CEI-009-20160601 (registration number R-2023-785-038). Between August 2023 and April 2025, a 0.5 mL aliquot was collected from samples scheduled for immunophenotypic evaluation using the EuroFlow panel for disease diagnosis or monitoring. These aliquots were stabilized in DNA/RNA Shield Solution (Cat. No. 1058, Zymo Research, Irvine, CA, USA).

The non-leukemia control group comprised pediatric bone marrow samples referred for immunophenotypic evaluation to rule out hematological malignancies. Using the standardized EuroFlow antibody panel, all samples were confirmed negative for leukemic blasts and did not meet diagnostic criteria for any hematological malignancy. Available clinical diagnoses in this group included hemophagocytic lymphohistiocytosis (n = 3), immune thrombocytopenia (n = 2), cytomegalovirus-associated mononucleosis syndrome (n = 1), and cytopenia of possible autoimmune etiology under diagnostic workup (n = 1). These conditions represent typical clinical scenarios requiring bone marrow evaluation to exclude leukemia in pediatric hematology practice.

A total of 178 bone marrow samples were processed.; RNA was extracted using the column-based Quick RNA Miniprep Plus Kit (Cat. No. 1058, Zymo Research, Irvine, CA, USA), and its concentration was quantified by spectrophotometry (260/280 nm). Subsequently, at least 250 ng of total RNA was reverse transcribed into cDNA using Oligo dT primers and the Transcriptor High Fidelity cDNA Synthesis Kit (Cat. No. 05 081 955 001, Roche, Basel, Switzerland).

### 4.2. Digital PCR Assays

Primers were engineered using the Primer-BLAST tool of the National Center for Biotechnology Information (https://www.ncbi.nlm.nih.gov/tools/primer-blast/index.cgi accessed on 20 August 2023) [53] and were synthesized by Integrated DNA technologies (Coralville, IA, USA). Gene sequences for primer design were obtained from the Gene Database of NCBI. Sequences are described in Table 2.

Each gene was amplified in separate reactions using the QIAcuity EvaGreen (EG) PCR Kit on a QIAcuity One instrument (QIAGEN, Venlo, Netherlands) over nanoplates with a resolution of 26,000 partitions. For inclusion in the analysis, samples were required to yield a minimum of 10,000 positive partitions for at least one of the reference genes (*RPS18* or *RPLP0*). Normalization of the target expression performed as follows:

(1) $$ositive\;partitions\;target\;\times \;\frac{\sqrt{Positive\;partitions\;RPS18\;\times Positive\;partitions\;\;RPLP0\;}}{\sqrt[{3}]{Valid\;partitions\;target\;\times \;Valid\;partitions\;RPS18\;\times Valid\;partitions\;RPLP0}}$$

### 4.3. Statistical Analysis

All statistical analysis were performed using Python, version 3.13, with standard scientific libraries, including scikit-learn, version 1.6.1, pandas, version 2.2.3, NumPy, version 2.3.4, Matplotlib, version 3.10.3, and Seaborn version 0.13.2. For individual marker evaluation, differences in gene expression across the four clinical groups were assessed by non-parametric KW test. Pairwise comparisons were conducted with the Mann–Whitney U test. Effect seizes for the KW test were calculated using eta-squared (η^2^) to quantify the magnitude of the association. Page’s trend test was applied to evaluate monotonic trends across clinical groups. All measured values, including zeros representing low or absent expression, were included in statistical analyses. The performance of individual markers in classifying two clinical states was evaluated using ROC curve analysis in four relevant comparisons. The AUC was calculated for each gene, and 95% confidence intervals were established from 1000 bootstrap resamples, with confidence boundaries defined by the 2.5th and 97.5th percentiles.

For multivariate analysis, the expression values of *JUP*, *NT5C3B*, *CNP*, *MYC*, *GATA3*, *PTK7*, *ICOSLG* and *SNAI1* were log2-transformed to normalize their exponential distribution. A subset of samples had missing gene expression data due to insufficient sample volume for dPCR reaction setup. The distribution of missing values across the genes was as follows: *JUP* and *NT5C3B* had no missing values (0/130, 0%). *CNP*, *ICOSLG* and *PTK7* (4/130, 3.08%), all from leukemia samples. *MYC* and *GATA3* (5/130, 3.85%), primarily from leukemia samples with one MRD-negative sample each. *SNAI1* (33/130, 25.38%) from leukemia (27/79), non-leukemia (3/11), and MRD-negative samples (3/29). These missing values were imputed using MICE with a Bayesian Ridge regression estimator, running for 10 iterations in ascending order.

The log2-transformed values were then standardized using Z-score normalization to ensure equal feature weighting. The Random Forest model was configured to 100 decision trees, balanced class weights, square root feature selection at each node split, and bootstrap sampling. This configuration allowed us to handle group size differences and ensure tree diversity. Model performance was evaluated using 5-fold cross-validation; the dataset was randomly split into five folds, with four used for training and one for validation, iterating until each fold served as the test once. Thus, prediction scores were generated for each patient using a model that had not been trained on their data. Furthermore, all preprocessing was performed separately within each cross-validation iteration to prevent information from the test set from influencing the training data. Feature importance was quantified by the mean decrease in Gini impurity. For the decision matrix visualization, principal component analysis was applied to the standardized data, retaining the first two components that explained the majority of the variance. The cross-validated AUC values from each fold were averaged to report mean performance with 95% confidence intervals. A confusion matrix was generated from the cross-validated predictions to calculate sensitivity, specificity and accuracy. To validate the added discriminatory value of the Random Forest approach over simpler methods, we also performed a baseline logistic regression model using the same 8-gene panel. The logistic regression model employed comparable preprocessing steps and was evaluated using the same 5-fold stratified cross-validation strategy. For all tests, an alpha of 0.05 was used to establish statistical significance.

## 5. Conclusions

This study validated an eight-gene panel (*JUP*, *CNP*, *NT5C3B*, *ICOSLG*, *SNAI1*, *PTK7*, *MYC*, and *GATA3*) for diagnosing and monitoring pediatric ALL using dPCR. A Random Forest classifier integrating this panel demonstrated robust performance, achieving 88.9% sensitivity and a mean cross-validated AUC of 0.908, significantly outperforming individual biomarkers.

This dPCR-based strategy addresses a critical need for quantitative, accessible molecular diagnostics that are independent of clonal heterogeneity. Its standardized workflow is especially advantageous for low-resource settings, where most pediatric cancers occur. Although this proof-of-concept study is promising, prospective validation in independent cohorts is essential. Future efforts will focus on panel optimization and functional investigation of newly identified targets like *NT5C3B* to further elucidate their biological and clinical relevance.

This study, conducted in a Mexican pediatric cohort from regions with high ALL incidence and resource constraints, demonstrates a proof-of-concept for accessible molecular diagnostics in resource-limited settings. As an easily implemented approach, this gene panel offers potential as a practical tool for initial disease detection. However, external validation in independent cohorts from diverse geographic and ethnic backgrounds will be essential to confirm generalizability and establish the panel’s utility across different populations and healthcare systems. Its role in therapeutic monitoring and risk stratification requires further investigation in prospective studies designed specifically for MRD assessment.

## Figures and Tables

**Table 1:** Evaluation of individual gene biomarkers for diagnostic classification. Optimal cutoff values, measured in normalized positive partitions, were determined for each gene by maximizing Youden index. This approach achieves an optimal balance between sensitivity and specificity for distinguishing between active disease (leukemia and MRD-positive patients) from disease absence or remission states (non-leukemia and MRD-negative patients). The Youden index, which ranges from 0 to 1, is calculated as the sum of sensitivity and specificity minus 1, with higher values indicating a more balanced diagnostic performance. Sensitivity represents the proportion of patients with active disease who are correctly identified (the true positive rate). Specificity indicates the proportion of individuals without the disease who are correctly classified (true negative rate). Overall accuracy reflects the percentage of all samples correctly classified regardless of disease status. Positive predictive value (PPV) represents the probability that a patient with a positive test truly has active disease, while negative predictive value (NPV) indicates the probability that a patient with a negative test result is truly free of disease. These metrics provide a comprehensive assessment of each gene’s diagnostic utility in clinical settings.

Gene	Optimal Cutoff	Youden Index	Sensitivity	Specificity	Accuracy	PPV	NPV
*PTK7*	3.413	0.520	0.657	0.864	0.708	0.936	0.452
*JUP*	11.246	0.491	0.691	0.800	0.719	0.906	0.480
*MYC*	5.119	0.463	0.845	0.618	0.780	0.845	0.618
*GATA3*	8.996	0.433	0.500	0.933	0.614	0.955	0.400
*NT5C3B*	31.582	0.422	0.622	0.800	0.677	0.875	0.485
*SNAI1*	2.893	0.420	0.587	0.833	0.656	0.900	0.441
*ICOSLG*	3.798	0.389	0.556	0.833	0.632	0.897	0.417
*CNP*	7.575	0.366	0.566	0.800	0.632	0.878	0.421

**Table 2:** Primer sequences and amplification conditions for dPCR analysis. Table displays forward (F’) and reverse (R’) primer sequences for the target and reference genes. The product sizes (PS) indicate the expected amplicon length in base pairs (bp), and the annealing temperature (T_a_) refers to the standardized temperature used for the annealing stage in the dPCR amplification protocol.

Gene	Name	NCBI Ref.	Sequence	PS (Pb)	T_a_ (°C)
*RPS18*	Ribosomal protein S18	NM_022551.3	F’ CGATGGGCGGCGGAAAA R’ CAGTCGCTCCAGGTCTTCACGG	283	60
*RPLP0*	Ribosomal protein lateral stalk subunit P0	NM_001002.4, NM_053275.4	F’ TGCATTCTCGCTTCCTGGAG R’ CCGACTCTTCCTTGGCTTCA	282	60
*JUP*	Junction plakoglobin	NM_001352775.2, NM_021991.4	F’ GCAGCCCTACACGGATG R’ ATGTTCTCCACCGACGAGT	160	58
*CNP*	2″,3″-cyclic nucleotide 3″ phosphodiesterase	NM_001330216.2, NM_033133.5	F’ AAGAAGGAGCTGCGACAA R’ GAAGGCCTTGGAGTAAGAT	190	60
*NT5C3B*	5′-nucleotidase, cytosolic IIIB	NM_052935.5, XM_047435298.1	F’ CAAGAACAGCTCTGCGTGTG R’ GCAGTAGCCCGTTGACCA	245	58
*GATA3*	GATA binding protein 3	NM_001002295.2, NM_002051.3	F’ AACCACACTCTGGAGGAGGA R’ ACGAGCTGTTCTTGGGGAAG	200	58
*BIRC5*	Baculoviral IAP repeat containing 5	NM_001168.3,	F’ GCTGGGAGCCAGATGACGACC R’ CGATGGCACGGCGCACTTT	210	58
*MYC*	MYC proto-oncogene	NM_002467.6, NM_001354870.1	F’ GAACTTACAACACCCGAGCAA R’ CAGTAGAAATACGGCTGCACC	249	58
*PTK7*	Protein tyrosine kinase 7	NM_152882.4, NM_001270398.2	F’ AACCGCTTTGTGCATAAGGA R’ CCCACATCAGCACACCGAAGG	223	58
*ICOSLG*	Inducible T cell costimulator ligand	NM_001365759.2, NM_001283052.2	F’ CTGTGCCTGCTTGTGGTCGTGGTG R’ TGGTGTGGATGACGCTTTTG	328	60
*SNAI1*	Snail family transcriptional repressor 1	NM_005985.4,	F’ CGAAAGGCCTTCAACTGCAA R’ CGCCTGGCACTGGTACTTCTT	270	64

## Data Availability

The data supporting the findings of this study are available within the article. For further inquiries, please contact the corresponding authors.

## Supplementary Materials

The following supporting information can be downloaded at: https://www.mdpi.com/article/10.3390/ijms27020674/s1.
