# Supplementary material for: Utility of a Digital PCR-Based Gene Expression Panel for Detection of Leukemic Cells in Pediatric Acute Lymphoblastic Leukemia
Source: Int J Mol Sci. 2026 Jan 9;27(2):674. doi: 10.3390/ijms27020674 (PMC12840621; doi:10.3390/ijms27020674)
Supplement: Supplementary file 1 [file ijms-27-00674-s001.zip › Supp Table S1.pdf]

Supplementary Table S1. Comparison of Random Forest and Logistic Regression models for active disease detection.

Metric	Random Forest	Logistic Regression
ROC AUC (mean ± SD)	0.908 ± 0.041	0.821 ± 0.033
PR AUC (mean ± SD)	0.961 ± 0.019	0.922 ± 0.016
Sensitivity	0.889	0.611
Specificity	0.650	0.850
Accuracy	0.815	0.685
PPV	0.851	0.902
NPV	0.722	0.486
