# Supplementary figures and images for: Utility of a Digital PCR-Based Gene Expression Panel for Detection of Leukemic Cells in Pediatric Acute Lymphoblastic Leukemia
Source: Int J Mol Sci. 2026 Jan 9;27(2):674. doi: 10.3390/ijms27020674 (PMC12840621; doi:10.3390/ijms27020674)

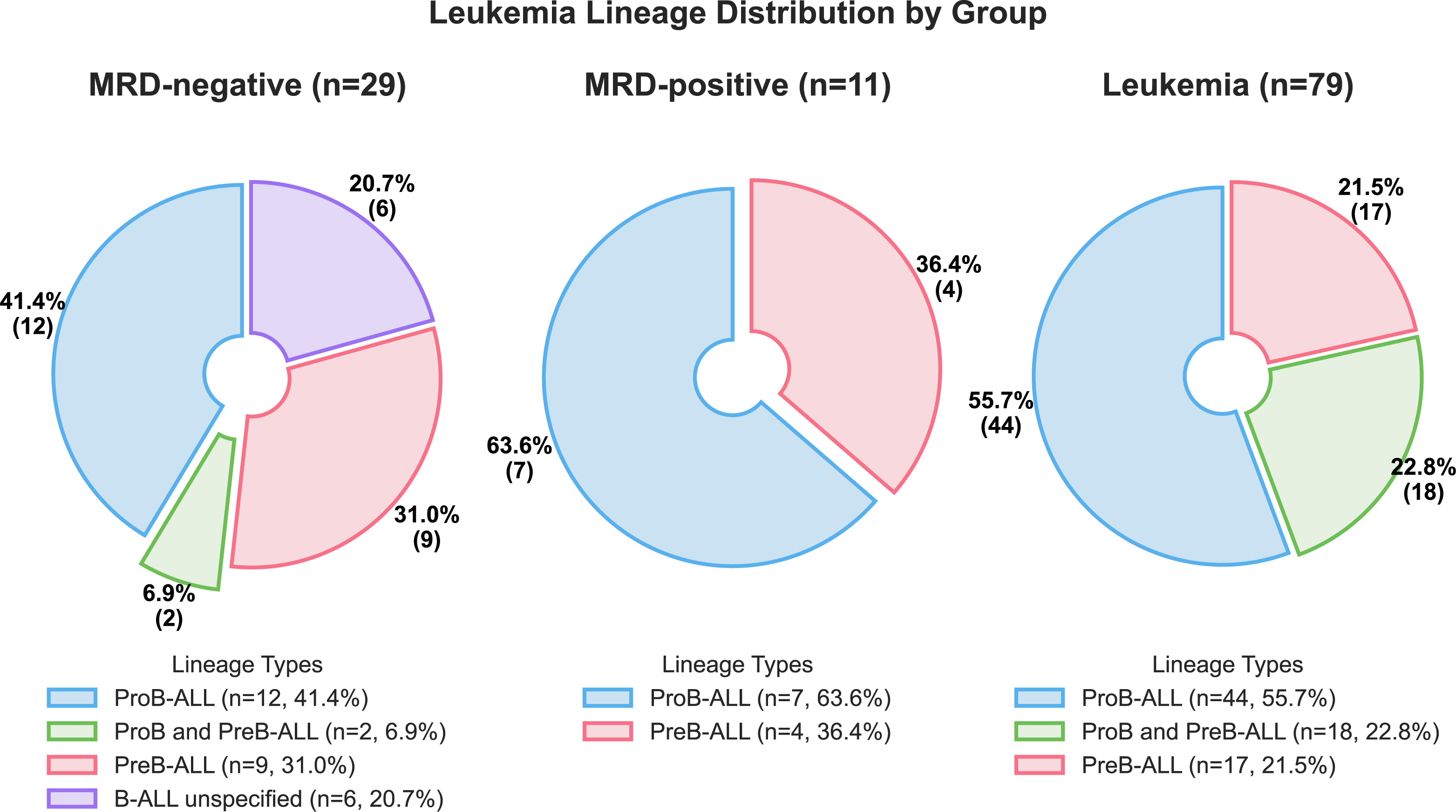

Supplement: Supplementary file 1 [file ijms-27-00674-s001.zip › Supp Figure S1.tif]

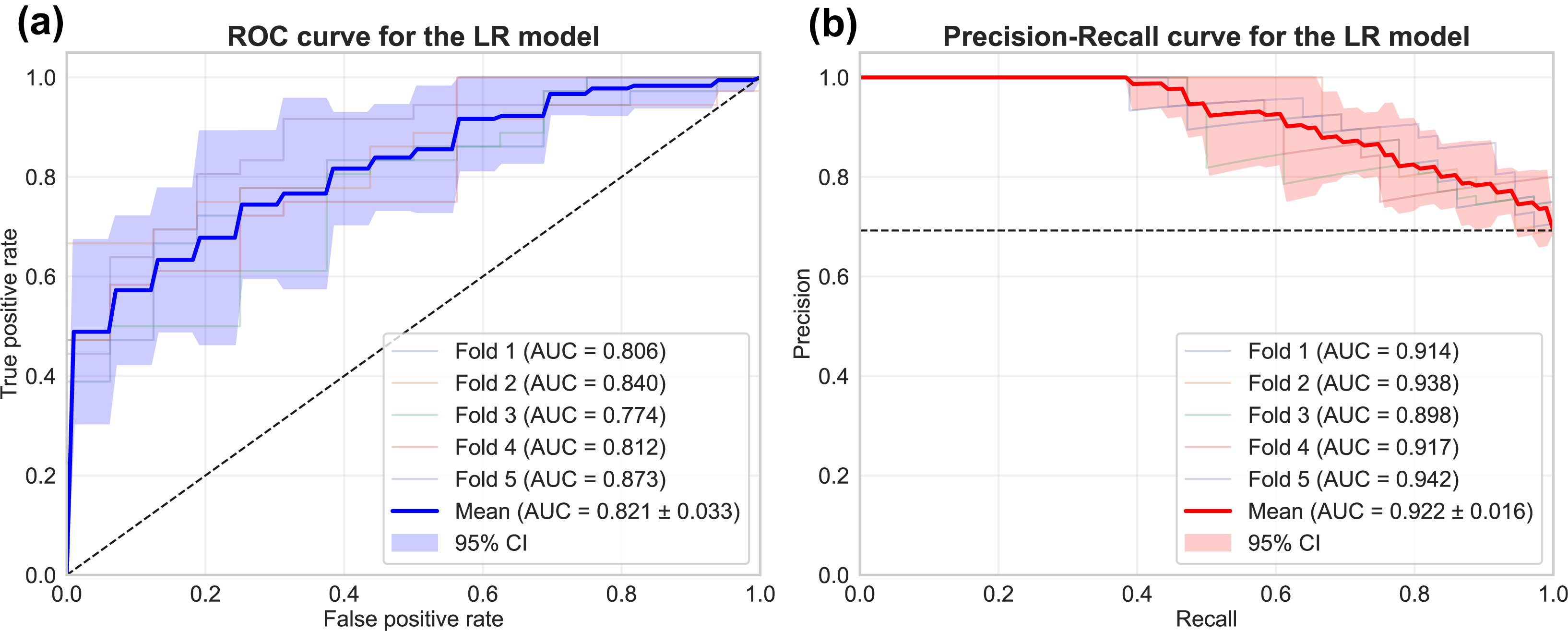

Supplement: Supplementary file 1 [file ijms-27-00674-s001.zip › Supp Figure S2.tif]
